# Report of a Case of Cancer of the Prostate

**Published:** 1887-12

**Authors:** W. T. Belfield


### REPORT OF A CASE OF CANCER OF THE PROSTATE.

ALSO, BY W. T. BELFIELD, M. D.

Hans J——, aged forty-eight years, was admitted to the Cook County Hospital, suffering from unduly frequent and somewhat painful urination, symptoms which had existed for some three months. On two occasions blood had been observed in the urine. Examination *per rectum* showed a slightly enlarged though symmetrical prostate, along the center of which, instead of the normal depression, was a slight protuberance. By the microscope we found in the urine a few blood corpuscles. A tumor of the prostate, either papillomatous or malignant, was diagnosed; the latter seemed more probable, because the quantity of blood in the urine was altogether too slight for the benignant variety. Efforts to entangle and bring away shreds of the supposed growth in the eye of a metallic catheter, failed. May 7th, the bladder was explored by supra-pubic incision, and a malignant villous growth was found growing from the left side of the prostate, the villi projecting an inch or more into the bladder. These were removed with forceps and spoon and the base cauterized. Recovery ensued without notable event, and for eight weeks the urinary function seemed almost normal; then the former symptoms began again and steadily increased in severity. The growth finally entirely filled the bladder and involved the abdominal wall, producing a small fistulous opening, and constituting a tumor which could be plainly outlined above the symphisis pubis and through the rectum, apparently filling the pelvis. Death occurred October 8th. Only the glands in the immediate vicinity of the bladder were found to be carcinomatous, and no secondary growths in other organs were discovered. The kidneys contained numerous miliary abscesses, a condition often consequent upon chronic cystitis.

